# The role of housing markets in producing respiratory health disparities

**DOI:** 10.1016/j.lana.2025.101228

**Published:** 2025-09-12

**Authors:** Evan Lemire, Sophia Carryl, S.V. Subramanian, Adam L. Haber

**Affiliations:** a Department of Environmental Health, Harvard T.H. Chan School of Public Health, Boston, MA, USA; b Department of Social and Behavioral Sciences, Harvard T.H. Chan School of Public Health, Boston, MA, USA; c FXB Center for Health & Human Rights, Harvard University, Boston, MA, USA

**Keywords:** Asthma, Housing, Health equity, Environmental justice, Indoor environment

## Abstract

Asthma is a chronic lung disease affecting an estimated 27 million people in the United States. It is much more prevalent and severe among Black, Hispanic, Indigenous, and working-class communities. Substandard housing disproportionately exposes poor people and communities of color to pests, mold, poorly regulated temperatures, and psychosocial stress. This role of housing as a driver of respiratory health disparities is well-established. However, less attention has been paid to the economic and political forces that produce the current distribution of substandard housing, which enables this unequal exposure and disease burden to persist. This review examines how harmful conditions arise from the political, legal, and economic relationships that govern housing’s production, maintenance, and distribution in the United States. We review emerging evidence that identifies power relations—most importantly the landlord-tenant relationship—as the motor that creates and reproduces disparities in exposures and the downstream disparities in asthma burden.

## Introduction

The right to healthy housing has been repeatedly affirmed in international declarations, beginning with the Universal Declaration of Human Rights signed by the United States in 1948.^1^ It is precisely the role of housing as a key determinant of health^2^^,^^3^ that these documents reference to justify its status as a universal right.^1^^,^^4^ Despite these commitments, millions of Americans are housed in inadequate and unhealthy homes or have no housing at all. In 2021, 6.7 million, 5.6% of all US households, were living in housing with substandard conditions, a figure that has held steady for the past two decades.^5^ In 2024, 771,800 people experienced homelessness, a record 18% increase over the prior year.^6^ This burden of unhealthy housing and housing insecurity is not shared equally; poor people and people of color are far more likely to live in substandard housing.^5^^,^^7^^,^^8^ This inequality has manifold implications for respiratory health, as these communities will be more likely to be exposed to pests, molds, and other risk factors for asthma because of the indoor environments they live in.

Despite the public health literature firmly linking housing and health, the social arrangements through which housing is produced and distributed—the housing market—is rarely, if ever, explicitly discussed. Where a resource is provided through a market, it is not available freely, but rather as a commodity for purchase or rent. Reynolds (2021)^9^ identifies commodification as a central pathway through which health inequalities are produced and maintained. Commodification creates the necessity of wealth for acquiring resources fundamental to health, and directly translates existing class stratification into health inequality. This selective availability applies not only to the most theorized health resource, health *care*, but also to other commodities essential for a healthy life, housing prominent among them.

Rental housing is central to the issue of housing and health. Renters account for more than one third of the US population, and are significantly more likely to live in substandard housing than non-renters.^5^ Renting as opposed to owning is associated with both greater asthma prevalence and severity.^8^^,^^10^ The landlord-tenant relationship is crucially important to the experience of rental housing, as the landlord typically bears responsibility for repairing poor or unhealthy conditions in the tenants building.^11^ To assess the extent to which landlord behavior factors into discussions of housing and respiratory health, we performed a PubMed search for “housing” AND “asthma” and retrieved 32 articles published since 2015. Of these, only 7 included any mention of landlords. A majority of those contained only a single mention, and in total the word “Landlord” appears 12 times across all 32 studies (Supplementary Table S1). Despite the central and direct role landlords play in determining housing conditions, none of the studies engaged in a substantive discussion of the landlord-tenant relationship.

**Fig. 1:**
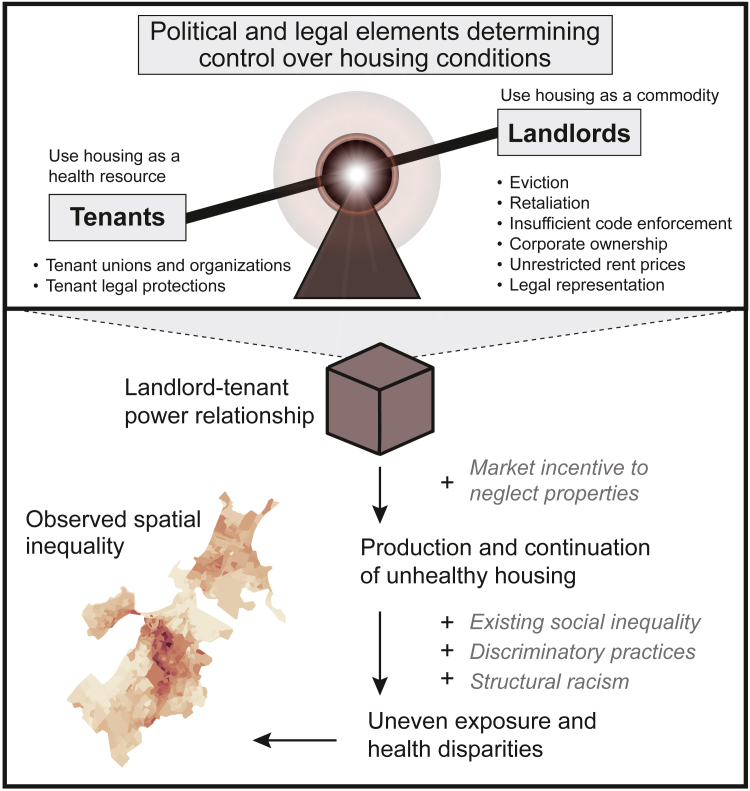
Power relationships inherent to market-based distribution of rental housing produce and maintain disparities in exposure to unhealthy housing: Conceptual illustration of the power relationship between landlords and tenants, and the elements of the balance of power within that relationship, as a core driver of respiratory health inequity. The second panel includes an illustrative map of spatial inequality showing the uneven distribution of rates of tenant reports of poor housing conditions across census block groups in the city of Boston.

This absence of this power relationship in discussions of housing and health is a crucial gap. In their review of the politics of housing, Madden and Marcuse observe that “the residential is political—which is to say that the outcome of the housing system is always the outcome of struggles between different classes and groups”.^12^ These relations between social groups have critical effects on the health and life chances of populations. The legal, economic, and political landscape of the rental market privileges landlord power to profit from housing as a commodity, at the expense of tenant power to use their home as a health resource. This landscape encompasses insufficient code enforcement, inadequate eviction protections, limited legal accountability for landlords, lack of legal representation for tenants, and scant if any restrictions on rental prices (Fig. 1).

In this review we first introduce the pathophysiology of asthma and mechanisms of indoor exposure-induced exacerbations, along with its prevalence and causes in the United States. We survey the literature on substandard housing as a causal factor in asthma pathogenesis and severity, as well as its role in producing different health outcomes by race and class. We then outline specific characteristics of the for-profit housing market that stratify exposure to dangerous conditions, despite the nominal rules in place to support access to healthy housing. We will discuss how a shift in power relations, and the consolidation of working-class power in institutions such as tenants’ unions, can lead to improved housing conditions by increasing the power of tenants over their homes. While this review is primarily focused on the political and legal context of housing in the United States of America, a majority of the world’s housing stock is privately owned^13^ and therefore other countries will share many of the same challenges and potential solutions as those detailed here.

**Search strategy and selection criteria.**

Original research references for this narrative review were selected from English language, peer reviewed articles using PubMed searches. Search terms for section 2 included: “Housing” AND “Asthma” OR “Respiratory Health” OR “Health disparities”. For section 3: “Substandard housing” AND “Health Inequity”. For section 4: “Substandard housing” AND “Limited Liability Corporations” OR “Code Enforcement”; “Health” AND “Eviction” OR “Rent burden”. For section 5: “Health” AND “Tenant Organizing”. References were selected from these results based on their relevance to the scope of this review.

## Indoor exposures from housing play a key role in asthma pathophysiology

Asthma is a chronic lung disease that currently affects around 8% of the US population^14^^,^^15^; it is characterized by shortness of breath and wheezing, persistent airway inflammation, and occasional exacerbations or ‘attacks’ that can be life threatening. It is similarly prevalent in adults and children, making it the most common chronic disease of childhood.^15^^,^^16^ Asthma prevalence and severity are highly disparate by race and class.^8^^,^^15^^,^^17^ In the United States, these health inequities are most pronounced in Black and Puerto Rican populations. Asthma prevalence is 34% higher among Black people of all ages than among white people, Black individuals are twice as likely as white individuals to have had a recent emergency department visit for asthma, and death from asthma among Black people is more than twice as likely as for white people.^15^ While there were initial attempts to identify genetic drivers of these disparities,^18^ these did not materialize, and a scientific consensus now attributes asthma disparities to environmental, social, and political determinants of health.^17^^,^^19^ Fitzpatrick et al. (2019),^20^ for instance, found that large racial disparities in asthma ED visit rates were completely explained by controlling for community and family level socioeconomic variables. Similar findings were reported by Alexander and Currie (2017),^21^ whose comprehensive analysis of state-wide data from every hospital visit in New Jersey from 2006 to 2012 (448,036 children), showed that after controlling for family characteristics and other confounders, the effect of neighborhood-level social variables completely explains Black–White disparities in childhood asthma.

The social and political forces that shape the physical environments where people live and work lead to differential exposure to asthma risk factors.^22^ The home is a primary source of relevant exposures, including allergens, toxicants, and stressors. Common household allergens, when inhaled, trigger immune sensitization and antibody production, leading to airway inflammation and hyperresponsiveness. More than 80% of children with asthma are sensitized to at least one indoor allergen, and this sensitization is a strong predictor of asthma development and persistence later in life.^23^ In addition to these allergens, the psychosocial stress associated with poor housing conditions can itself contribute to asthma exacerbation.^24^ A large meta-analysis of 227 studies^25^ showed that indoor triggers, particularly mold, are the strongest risk factors for asthma with the exception of respiratory viral infection, which can itself be strongly influenced by housing if residents are crowded.^26^ Reviews of the literature by Heinrich^27^ and Dick et al.^28^ confirm these findings, describing a range of randomized control trials that again emphasize the central role of mold. In addition to mold, the presence of household pests, including cockroaches, mice, and dust mites, is strongly associated with childhood asthma.^29^^,^^30^ Several studies shown that moving from poor quality housing into better quality housing results in improved respiratory outcomes, demonstrating a causal relationship between housing quality and asthma. Colton et al. (2015) showed that children who moved from conventional to ‘green’ public housing units with improved ventilation, fewer pests, and less mold, experienced 3-fold fewer asthma attacks, 4-fold fewer hospitalizations, and almost 5-fold fewer missed school days.^31^ A similar study based in Chicago found that 65% of asthmatic children who moved into “green” public housing reported improved asthma outcomes and were significantly less likely to need to use a prescription inhaler than control group asthmatic children.^32^

## Markets produce unequal distributions of healthy housing

There is substantial evidence that the burden of poor housing in the United States is not shared equally. The most comprehensive data on national housing quality, the American Housing Survey, has found that the rental market bears a disproportionate burden of poor housing. Renters are 2.2 times as likely to live in moderately substandard and 2.7 times as likely to live in severely substandard housing as homeowners.^33^ Poor housing is also unequally distributed by race and class. Black Americans are 1.9 times as likely to live in moderately substandard housing, and 2.7 times as likely to live in severely substandard housing, as their white counterparts (2023 estimates).^7^^,^^33^ Households with incomes below the federal poverty threshold were 2 times as likely to live in moderately substandard, and 3.1 times as likely to live in severely substandard housing as those above the federal poverty threshold.

Adding to these survey data, tenant reports of poor conditions to city authorities provide a further line of evidence of racial and class disparities in housing conditions. Our previous work^34^ found that the strongest predictors of neighborhood incidence of tenant reports for asthma triggers in Boston, MA were the proportion of white residents and median household income of a census block group. Beck et al. (2014)^35^ identified an association between the density of housing code violations and emergency department usage by asthmatic children in Cincinnati. Taken together, these data reveal that for-profit housing markets produces a distribution of housing quality that strongly varies by race and class, making it an important determinant of exposure and ultimately health disparities.

## Regulatory structures facilitate landlord power

Given these unequal and deleterious outcomes, further investigation of specific social, economic, and political structures that determine access to quality housing is needed. In rental housing, the legal structures specifying the relationship between landlords and tenants are particularly critical. Examples of these structures include reactive complaint-based systems that result in insufficient and unequal code enforcement, corporate entities that limit landlord exposure to legal and financial liability, lack of legal representation for tenants, inadequate restrictions on rental burdens, and, crucially, the power of landlords to evict tenants from their homes.

### Existing tenant laws provide insufficient protection

In most US jurisdictions, it is the landlord’s legal responsibility to maintain livable housing, and municipal housing codes are the main enforcement mechanism used by local governments.^11^ Enforcement is almost exclusively ‘complaint-based’, in that in the event of a deleterious exposure or conditions, tenants must contact regulatory agencies to request an inspection to verify the problem and potentially cite the landlord for a housing code violation. This arrangement is notorious for neglecting marginalized populations, who may not report unhealthy conditions, either because they fear retaliation from landlords and law enforcement or because the systems to do so are not disseminated widely enough or in the appropriate language.^36^ Compounding this situation, the municipal response to complaints also may vary by neighborhood, social class, and racial demographics, further contributing to health inequity. We previously demonstrated that the municipal response to tenant reports in Boston is markedly slower and less effective in poorer and less white census block groups.^34^

Tenants in all US states except Arkansas have further recourse to abate poor conditions through implied warranty of habitability laws, which allow renters to withhold rent if certain conditions are not met.^37^ However, exercising this legal protection means tenants risk landlord retaliation and must hire a lawyer, which is unlikely to be a viable option for marginalized and low-income tenants. The shortcomings of this nominal legal protection were confirmed by a recent study which found no change in housing-related health conditions after the introduction of implied warranty of habitability laws across nine states. Thus, existing tenant laws, most prominently municipal housing codes and implied warranty of habitability laws, which typically place extensive requirements of time, money, and risk on tenants, are insufficient to protect tenant health unless strengthened or paired with additional protections such as tenants right to counsel.^38^

### Limited liability companies (LLCs) shield landlords from accountability

Corporate structures that shield landlords from legal liability can potentially change landlord behavior when it comes to maintaining their properties by diminishing vulnerability to regulatory structures designed to keep tenants safe, and to direct legal action by tenants. A particularly attractive corporate form for landlords who may face legal liability or fines for improper upkeep of their properties is the LLC. LLCs are corporate entities that offers the liability protection of a standard corporation without the associated tax burden.^39^ Beginning in Wyoming in 1977, and accelerating rapidly in the 1990s, state legislation allowed for the use of LLCs in housing ownership.^40^ They are now in use nationwide. As of 2020 more than 15% of the nation’s rental properties, and more than 40% of rental units, are owned by an LLC or similar entity.^41^ Horner (2019)^39^ points out that landlords often use many different LLCs, sometimes one per property. Since in most jurisdictions, landlords can still evict tenants even if their property is in disrepair, increased use of single-property LLCs could potentially lead to increased rates of neglect and increased eviction, especially for low-income tenants.^39^ A quasi-experimental study in Milwaukee^42^ confirmed this is indeed the case, finding substantially higher code violation rates in buildings after conversion to LLC ownership.

### High rental and housing costs erode tenant power and directly impact their health

In recent decades, rent prices in the United States have dramatically increased while wages have remained stagnant. In 2015, for example, the average proportion of total income that poor renters spent on rent was 11 percentage points higher than their 2000 counterparts.^43^ As of 2018, a majority of families below the federal poverty line spent more than half of their income on housing costs.^44^ This high expenditure on rent means less available money for other health necessities, less than $500 each month for renters in the bottom quintile of the income distribution,^43^ a dire situation for renter health. Emphasizing this point, Graetz (2024)^45^ finds that high rent burden is associated with substantial increases in all-cause mortality.

Improperly maintained housing also increases total housing costs by creating energy inefficiencies. Substandard housing is more difficult to properly heat and cool, leading to elevated energy costs and increased exposure to extreme temperatures for low income tenants.^46^ This added financial stress may engender coping strategies such as underutilization of heating systems, or usage of dangerous alternatives such as ovens, and together the increased exposure to improper temperatures and harmful exposures due to energy hardship has major implications for tenant respiratory health.^47^ In this way, landlords offset the costs of housing maintenance onto their tenants either financially, or in terms of their health.

In addition to the direct effects of high housing costs on health, high rents also weaken tenant power in relation to landlords and the overall landscape of the housing market. Tenant insecurity is intensified when alternative housing is difficult to find due to overall high rental costs.^48^ Faced with the daunting prospect of acquiring a place to live in an expensive rental market, tenants may be less likely to speak up for their rights related to healthy housing, such as requesting repairs, or calling for a municipal code enforcement inspection, for fear that it may bring them into conflict with their landlord and jeopardize their housing.

An additional indicator of the deleterious effects of high rents on exposure to poor housing conditions is that, since 2000, overall housing quality has not improved.^44^ Vacancy rates have increased on average, but in the cheapest part of the market, they have substantially decreased.^44^ Together, this may indicate that increasing rents are pushing a greater number of tenants into cheaper, lower quality housing, potentially exposing them to greater health risks. Most importantly, without any limit on rents, landlords are able to increase rent prices beyond a level that the tenant can afford. This can result either in informal evictions where tenants elect to move rather than sign a new lease at a rent they can’t afford, or if they fall behind the landlord may file formal eviction proceedings.

Finally—high rental burden impacts respiratory health by increasing risk of losing access to housing. Inability to pay rent due to rising costs constitutes a significant pathway to homelessness.^49^ Rates of homelessness have been shown to rise faster where rental burden is higher, and rises in rent levels precede rises in rates of homelessness.^50^ The experience of homelessness is profoundly disruptive, leading to psychosocial stress and increased environmental exposures. Consistent with this, children who have ever experienced homelessness are at greater risk of developing asthma.^51^

## Evictions have manifold deleterious effects on tenant health

Forced moves, or formal evictions, are a critical aspect of the distribution of housing via a for-profit market, that enable landlords to terminate rental agreements where tenants cannot afford the rent. Eviction filings–where a landlord submits documents to housing court to initiate the eviction process–are the start of a protracted legal process that, if a judge rules against the tenant, may end in forced removal of the tenant and their belongings by the police. The ability of landlords to evict tenants who can’t afford the rent is a key expression of housing as a commodity^9^ where access to a critical health necessity is determined by an individual’s ability to pay. In Boston, approximately 80% of evictions are for non-payment of rent.^52^ The risk of housing instability and homelessness caused by eviction has clear and significant health implications, including higher rates of chronic diseases, substance abuse, and all-cause mortality.^53^ But the process of eviction itself also produces its own health risks even if tenants can find new housing afterwards.

Eviction proceedings have serious effects on the health of tenants, resulting in increased emergency department usage, and severe stress.^54^^,^^55^ Graetz (2024)^45^ found eviction filings alone were associated with a 19% increase in all-cause mortality, and *eviction judgements with a 40% increase*. These deleterious health effects also fall hardest on racialized tenants. Despite making up 13% of the population, and 18% of renters, Black tenants account for 51.1% of all eviction filings.^56^ In light of this, it is clear that evictions are a major contributor to racial health disparities. Beyond all-cause mortality, there is a relative paucity of research investigating the link between eviction and respiratory outcomes, though there is reason to expect that eviction may contribute to increased risk of asthma exacerbations effect in two ways. First, after an eviction, assuming they can find housing, tenants may be more likely to move into sub-standard or precarious housing.^54^^,^^57^ Second the associated psychosocial stress may directly increase the risk of asthma exacerbation via neuroimmune mechanisms.^58^ More investigation is needed to definitively establish the link between eviction and asthma exacerbations. For example, cohort studies of asthma outcomes after evictions, analysis of emerging data sources such as smart inhalers^59^ or quasi-experimental analyses of major changes in eviction policies or economic events, could all offer promising directions to expand this research literature.

Lastly, eviction can also be an ever-present threat hanging over the personal relationship between landlord and tenant. Tenants know they could face retaliatory eviction if their relationship with their landlord sours, and this fear of retaliation may stop a tenant from filing complaints about the poor condition of their housing. A mixed methods interview study of participants in the Massachusetts Breathe Easy at Home program, which allows clinicians to request inspections on behalf of asthma patients if they deem that housing conditions are a contributing factor, found that many tenants described reluctance to participate in the program for fear of landlord retaliation.^60^ Landlord retaliation is nominally prohibited in 41 states and the District of Columbia.^11^ In Massachusetts, for example, when a tenant has exercised their rights to request repairs or organize a tenants union, including filing a complaint about the condition of their housing, eviction proceedings or rent increases within 6 months are assumed to be retaliatory.^61^ However, these protections are limited by the fact that retaliation must be argued in court. Marginalized tenants may move rather than face housing court, and where they do attend housing court, tenants are substantially less likely than landlords to be represented by a lawyer. Nationwide, approximately 90% of landlords come to housing court with legal representation, only 10% of tenants have access to a lawyer.^62^

## Tenant organizing and collective action can change the balance of power

The relationship between landlords and tenants is defined by manifold differences in power that frequently pit the landlord’s profit against the tenants’ health. Facing this political landscape, there is a long history of tenants, particularly in low-income Black and Latino neighborhoods, organizing political campaigns for better housing conditions. Asthma in particular has become a signature environmental health issue for urban communities of color,^63^ who have long faced elevated exposure to bad housing conditions. In 1935, the poet Langston Hughes described these struggles in Harlem, writing that ‘equality is in the air we breathe’. Clean air and clean housing have been central demands of social movements led by people of color in the U.S., from the Black Panthers 10-point program^64^ that called for ‘decent housing, fit for the shelter of human beings’, to the Puerto Rican Young Lords who in 1970 held a mock trial of landlords and city officials in the “People’s Court for Housing Crimes”.^65^ Recent years have seen a newly resurgent tenant movement, with tenant organizations and unions springing up across the country.^66^ Michener (2023)^67^ recently reviewed how these tenant organizations fight for improvements in the condition of their housing as a primary, if not the only, mechanism tenants have to work towards health equity. At the grassroots, tenants in individual buildings collectively agitate for repairs to build direct and also public pressure on their landlord, while city or statewide tenant movements push for legislative reforms to shift the balance of tenant and landlord power. Tenants unions have won improved conditions in health-relevant housing conditions across the US, including the recent federally ordered lead abatement fought for by tenants in an apartment building in Putnam, Connecticut,^68^ or the 75 Prospect Street tenants association in New Jersey which had their building placed in receivership by a county judge due to persistent mold issues in the building.^69^ Dampness and mold abatement have also been the subject of successful union fights in Palm Beach, FL^70^ New Haven CT,^71^ and Omaha, NE.^72^

In addition to winning repairs of market rate housing, tenant movements have also fought for expansions of public housing, or against its demolition, and against the displacing effects of gentrification.^73^ While government owned and operated housing, when properly funded, solves the issue of market incentive to neglect, the provision of public housing has unfortunately been decimated in the last half century. Public housing stock has dropped from a one time national high of 1.5 million units in 1994^74^ to just over 900,000 today.^75^ As the public housing stock has been reduced, it has been replaced by a range of public-private partnerships which, at bottom, suffer the same underlying issues common to the private housing market.^74^ Recognizing this, tenant and social movements have fought for the expansion and repair of public housing, and these demands continue today.^65^^,^^73^ Even in the private market, tenant movements can be an effective bulwark against the process of gentrification—whereby new development can lead to an increase in rental prices and displacement of the original residents. Tenants movements have organized successfully against rent increases and associated displacement in their communities, such as the recent tenant union campaign at a 350-unit housing complex in Boston’s Mattapan neighborhood which won rents capped at a maximum of 2% annual increases.^76^ Where successful, tenant-led campaigns directly protect the health of these communities by mitigating the effects of higher rental burdens and poor housing conditions on tenant health.

Tenant movements constitute an important site of power for tenants that can tip the scales away from housing as a commodity and towards housing as a necessity for health. In light of this, identifying and promoting these mechanisms of tenant power to access and control stable, healthy housing is essential to promote respiratory health and reduce disparities. Tenant organizing efforts have been galvanized by expanded tenant protection legislation, and in particular, legal recognition of tenants’ rights to organize. The City of New Haven, Connecticut, for instance, signed an ordinance in 2022 recognizing the rights of tenants unions to collectively bargain with the support of city government and a surge of tenants union formation and organizing followed.^77^^,^^78^ In the same year San Francisco passed a ‘union at home’ ordinance requiring landlords to bargain in good faith with tenant unions; unionization of over 1100 housing units in more than 60 building-level unions followed.^79^ Such laws protecting the right to organize as tenants are emerging mechanisms to support the formation of tenant associations, expanding the reach of benefits that they have for population health. The effects that tenants unions have on health merits further study in the public health literature. As of yet, no studies have investigated what effect tenant union membership or supportive legislation, such as the New Haven ordinance, has on tenant respiratory health, and there is potential and a clear need for community-engaged public health research to further understand and support these efforts.^63^

## Conclusion

Public health literature has established housing as a critical social determinant of health, particularly for respiratory diseases such as asthma. Despite this extensive evidence base, large segments of the population in the US and worldwide^80^ do not live in healthy homes, and substantial health inequities are produced as a result. Addressing the question of why these obvious injustices persist requires researchers to investigate the system through which housing is distributed: the housing market, and the major classes of actors within those markets—landlords and tenants. The commodification of housing and its associated political, legal, and social landscape is in direct conflict with housing’s role as a necessity for healthy lives. It should be stressed that this policy landscape is a reflection not only of missing knowledge—widespread poor housing conditions have persisted long after its links to health have been established and widely disseminated—but of the balance of power between different social classes.^9^^,^^81^ Landlords and other sectors (such as real estate agents, developers, property managers, etc.) who profit from commodified housing are organized into advocacy organizations and lobby groups, which typically fight against the passage of legislation that would intervene to protect tenants.^82^ On the other side, tenants unions have won improved housing conditions in buildings across the country,^67^ but not enough research has yet been done on the health benefits of membership in such organizations.

Public health research that quantifies the effects tenant union membership on respiratory and other health outcomes is needed to better characterize their potential to prevent harmful exposures. Research is also needed to identify the key aspects of legal power that landlords exercise over tenant health to inform effective policies for tenant protection. Such interventions include removing loopholes landlords use to avoid legal liability, stronger and more proactive code enforcement measures, and ensuring tenants have a right to legal counsel in housing court, where the vast majority currently go without.^62^ Analyzing the specific power relations that underly and produce health inequities is the necessary first step to guide research questions asked, data collected, and solutions put forward. Without these insights, studies obfuscate the actual processes affecting health and therefore cannot address the root cause of housing related health conditions, which is ultimately necessary to ensure that everyone lives in a healthy home. Public health research that examines these relations of class power that underly observable health disparities enables researchers, tenants, and other political actors to produce and engage with evidence in a way that can catalyze changes in our social structure and work toward a healthier, more just world.

## Contributors

Evan Lemire, BS: Conceptualization, literature search, figures, writing–original draft, writing–reviewing and editing.

Sophia Carryl, PhD: Writing–reviewing and editing.

S.V. Subramanian, PhD: Writing–reviewing and editing.

Adam L. Haber, PhD: Conceptualization, figures, writing, reviewing and editing, decision to submit.

## Declaration of interests

We have no competing interests.
