# Supplementary material for: The role of housing markets in producing respiratory health disparities
Source: Lancet Reg Health Am. 2025 Sep 12;51:101228. doi: 10.1016/j.lana.2025.101228 (PMC12664648; doi:10.1016/j.lana.2025.101228)
Supplement: Supplementary Table S1 [file mmc1.docx]

**The role of housing markets in producing respiratory health disparities**

**Supplementary materials**

**Supplementary Table 1**: Results of literature search regarding discussion of landlord-tenant relationships in research on housing and respiratory health.

| **Citation** | **Mentions of "Landlord"** | **Mentions of "Owner"** | **Discussion of landlord-tenant relationship?** |
| --- | --- | --- | --- |
| [Abebe, Y., Ali, A., Kumie, A., Haile, T., Tamire, M., & Addissie, A. (2021). Determinants of asthma in Ethiopia: Age and sex matched case control study with special reference to household fuel exposure and housing characteristics. Asthma Research and Practice, 7(1), 14. https://doi.org/10.1186/s40733-021-00080-2](https://doi.org/10.1186/s40733-021-00080-2) | 0 | 0 | N |
| [Alcala, E., Capitman, J. A., & Cisneros, R. (2023). The moderating role of housing quality on concentrated poverty and asthma-related emergency department visits among Hispanics/Latinos. Journal of Asthma, 60(10), 1816–1823. https://doi.org/10.1080/02770903.2023.2188567](https://doi.org/10.1080/02770903.2023.2188567) | 0 | 0 | N |
| [Beck, A. F., Wymer, L., Pinzer, E., Friedman, W., Ashley, P. J., & Vesper, S. (2023). Reduced prevalence of childhood asthma after housing renovations in an underresourced community. Journal of Allergy and Clinical Immunology: Global, 2(4), 100143. https://doi.org/10.1016/j.jacig.2023.100143](https://doi.org/10.1016/j.jacig.2023.100143) | 0 | 0 | N |
| [Bryant-Stephens, T. C., Strane, D., Robinson, E. K., Bhambhani, S., & Kenyon, C. C. (2021). Housing and asthma disparities. Journal of Allergy and Clinical Immunology, 148(5), 1121–1129. https://doi.org/10.1016/j.jaci.2021.09.023](https://doi.org/10.1016/j.jaci.2021.09.023) | 1 | 0 | N |
| [Byun, J., McDonnell, S., & Robertson, J. (2019). Using Asthma-Related Housing Complaints to Target Residents With Uncontrolled Asthma in Salt Lake County, Utah. Preventing Chronic Disease, 16, 180463. https://doi.org/10.5888/pcd16.180463](https://doi.org/10.5888/pcd16.180463) | 0 | 0 | N |
| [Chevereau-Choquet, M., Thoreau, B., Taillé, C., Marchand-Adam, S., Morel, H., Plantier, L., & Portel, L. (2024). Smoking, Urban Housing and Work-Aggravated Asthma are Associated with Asthma Severity in a Cross-Sectional Observational Study. Journal of Asthma and Allergy, Volume 17, 69–79. https://doi.org/10.2147/JAA.S424546](https://doi.org/10.2147/JAA.S424546) | 0 | 0 | N |
| [Christian, W. J., Flunker, J., May, B., Westneat, S., Sanderson, W. T., Schoenberg, N., & Browning, S. R. (2023). Adult asthma associated with roadway density and housing in rural Appalachia: The Mountain Air Project (MAP). Environmental Health, 22(1), 28. https://doi.org/10.1186/s12940-023-00984-x](https://doi.org/10.1186/s12940-023-00984-x) | 0 | 0 | N |
| [Colton, M. D., Laurent, J. G. C., MacNaughton, P., Kane, J., Bennett-Fripp, M., Spengler, J., & Adamkiewicz, G. (2015). Health Benefits of Green Public Housing: Associations With Asthma Morbidity and Building-Related Symptoms. American Journal of Public Health, 105(12), 2482–2489. https://doi.org/10.2105/AJPH.2015.302793](https://doi.org/10.2105/AJPH.2015.302793) | 0 | 1 | N |
| [Faison, K., Moon, A., Buckman, C., Cortright, L., Tumin, D., Campbell, C., & Beamon, B. (2021). Change of address as a measure of housing insecurity predicting rural emergency department revisits after asthma exacerbation. Journal of Asthma, 58(12), 1616–1622. https://doi.org/10.1080/02770903.2020.1818773](https://doi.org/10.1080/02770903.2020.1818773) | 0 | 0 | N |
| [Gabbay, J. M., Abrams, E. M., Nyenhuis, S. M., & Wu, A. C. (2023). Housing Insecurity and Asthma Outcomes. The Journal of Allergy and Clinical Immunology: In Practice, S2213219823011480. https://doi.org/10.1016/j.jaip.2023.10.031](https://doi.org/10.1016/j.jaip.2023.10.031) | 3 | 0 | N |
| [Gabbay, J. M., & Wu, A. C. (2023). Housing Mobility Intervention and the Impact on Pediatric Asthma Morbidity: A Novel Asthma-Directed Therapy. The Journal of Allergy and Clinical Immunology: In Practice, 11(8), 2622–2623. https://doi.org/10.1016/j.jaip.2023.06.020](https://doi.org/10.1016/j.jaip.2023.06.020) | 0 | 0 | N |
| [Howard, A., Mansour, A., Warren-Myers, G., Jensen, C., & Bentley, R. (2023). Housing typologies and asthma: A scoping review. BMC Public Health, 23(1), 1766. https://doi.org/10.1186/s12889-023-16594-8](https://doi.org/10.1186/s12889-023-16594-8) | 0 | 0 | N |
| [Hughes, H. K., Matsui, E. C., Tschudy, M. M., Pollack, C. E., & Keet, C. A. (2017). Pediatric Asthma Health Disparities: Race, Hardship, Housing, and Asthma in a National Survey. Academic Pediatrics, 17(2), 127–134. https://doi.org/10.1016/j.acap.2016.11.011](https://doi.org/10.1016/j.acap.2016.11.011) | 0 | 0 | N |
| [Kearney, G. D., & Kuranga, A. O. (2017). Moving Upstream on Childhood Asthma and Housing. Journal of Public Health Management and Practice, 23(2), 187–191. https://doi.org/10.1097/PHH.0000000000000532](https://doi.org/10.1097/PHH.0000000000000532) | 4 | 0 | N |
| [Kim, B., Mulready-Ward, C., Thorpe, L. E., & Titus, A. R. (2022). Housing environments and asthma outcomes within population-based samples of adults and children in NYC. Preventive Medicine, 161, 107147. https://doi.org/10.1016/j.ypmed.2022.107147](https://doi.org/10.1016/j.ypmed.2022.107147) | 0 | 0 | N |
| [Knibbs, L. D., Woldeyohannes, S., Marks, G. B., & Cowie, C. T. (2018). Damp housing, gas stoves, and the burden of childhood asthma in Australia. Medical Journal of Australia, 208(7), 299–302. https://doi.org/10.5694/mja17.00469](https://doi.org/10.5694/mja17.00469) | 0 | 0 | N |
| [Liao, W., Zhou, L., Zhao, X., Song, L., Lu, Y., Zhong, N., Yang, P., Sun, B., & Zhang, X. (2017). Thermoneutral housing temperature regulates T-regulatory cell function and inhibits ovabumin-induced asthma development in mice. Scientific Reports, 7(1), 7123. https://doi.org/10.1038/s41598-017-07471-7](https://doi.org/10.1038/s41598-017-07471-7) | 0 | 0 | N |
| [Mazenq, J., Dubus, J.-C., Gaudart, J., Charpin, D., Viudes, G., & Noel, G. (2017). City housing atmospheric pollutant impact on emergency visit for asthma: A classification and regression tree approach. Respiratory Medicine, 132, 1–8. https://doi.org/10.1016/j.rmed.2017.09.004](https://doi.org/10.1016/j.rmed.2017.09.004) | 0 | 0 | N |
| [Mehta, A. J., Dooley, D. P., Kane, J., Reid, M., & Shah, S. N. (2018). Subsidized Housing and Adult Asthma in Boston, 2010–2015. American Journal of Public Health, 108(8), 1059–1065. https://doi.org/10.2105/AJPH.2018.304468](https://doi.org/10.2105/AJPH.2018.304468) | 1 | 0 | N |
| [Moses, L., Morrissey, K., Sharpe, R. A., & Taylor, T. (2019). Exposure to Indoor Mouldy Odour Increases the Risk of Asthma in Older Adults Living in Social Housing. International Journal of Environmental Research and Public Health, 16(14), 2600. https://doi.org/10.3390/ijerph16142600](https://doi.org/10.3390/ijerph16142600) | 0 | 0 | N |
| [Oshikata, C., Watanabe, M., Ishida, M., Kobayashi, S., Hashimoto, K., Kobayashi, N., Yamazaki, A., Konuma, R., Kaneko, T., Kamata, Y., Kuriyama, S., Yanai, M., & Tsurikisawa, N. (2021). Association between Temporary Housing Habitation after the 2011 Japan Earthquake and Mite Allergen Sensitization and Asthma Development. International Archives of Allergy and Immunology, 182(10), 949–961. https://doi.org/10.1159/000515870](https://doi.org/10.1159/000515870) | 0 | 0 | N |
| [Oshikata, C., Watanabe, M., Ishida, M., Kobayashi, S., Hashimoto, K., Kobayashi, N., Yamazaki, A., Konuma, R., Shimada, T., Kaneko, T., Kamata, Y., Kuriyama, S., Yanai, M., & Tsurikisawa, N. (2021). A mite allergen avoidance decreased mite-specific IgE levels and ameliorated asthma symptoms in subjects who lived in temporary housing after natural disasters. Allergologia et Immunopathologia, 49(4), 171–179. https://doi.org/10.15586/aei.v49i4.240](https://doi.org/10.15586/aei.v49i4.240) | 0 | 0 | N |
| [Perovich, L. J., Ohayon, J. L., Cousins, E. M., Morello-Frosch, R., Brown, P., Adamkiewicz, G., & Brody, J. G. (2018). Reporting to parents on children’s exposures to asthma triggers in low-income and public housing, an interview-based case study of ethics, environmental literacy, individual action, and public health benefits. Environmental Health, 17(1), 48. https://doi.org/10.1186/s12940-018-0395-9](https://doi.org/10.1186/s12940-018-0395-9) | 0 | 0 | N |
| [Pollack, C. E., Roberts, L. C., Peng, R. D., Cimbolic, P., Judy, D., Balcer-Whaley, S., Grant, T., Rule, A., Deluca, S., Davis, M. F., Wright, R. J., Keet, C. A., & Matsui, E. C. (2023). Association of a Housing Mobility Program With Childhood Asthma Symptoms and Exacerbations. JAMA, 329(19), 1671. https://doi.org/10.1001/jama.2023.6488](https://doi.org/10.1001/jama.2023.6488) | 1 | 0 | N |
| [Sandel, M. T., & Bovell-Ammon, A. (2020). Associations Between Federal Rental Housing Assistance and Childhood Asthma—A Renewed Call for Investing in Housing for Health. JAMA Pediatrics, 174(6), 525. https://doi.org/10.1001/jamapediatrics.2019.6272](https://doi.org/10.1001/jamapediatrics.2019.6272) | 1 | 0 | N |
| [Shiue, I. (2015). Indoor mildew odour in old housing was associated with adult allergic symptoms, asthma, chronic bronchitis, vision, sleep and self-rated health: USA NHANES, 2005–2006. Environmental Science and Pollution Research, 22(18), 14234–14240. https://doi.org/10.1007/s11356-015-4671-8](https://doi.org/10.1007/s11356-015-4671-8) | 0 | 0 | N |
| [Thakur, N., & Martinez, A. (2023). Housing Reparations as an Avenue to Counter the Impact of Structural Racism on Asthma. JAMA, 329(19), 1645. https://doi.org/10.1001/jama.2023.7242](https://doi.org/10.1001/jama.2023.7242) | 0 | 0 | N |
| [Tieskens, K. F., Milando, C. W., Underhill, L. J., Vermeer, K., Levy, J. I., & Fabian, M. P. (2021). The impact of energy retrofits on pediatric asthma exacerbation in a Boston multi-family housing complex: A systems science approach. Environmental Health, 20(1), 14. https://doi.org/10.1186/s12940-021-00699-x](https://doi.org/10.1186/s12940-021-00699-x) | 1 | 1 | N |
| [Titus, A. R., Terlizzi, K., Conderino, S., Ðoàn, L. N., Kim, B., & Thorpe, L. E. (2024). Patterns and drivers of disparities in pediatric asthma outcomes among Medicaid-enrolled children living in subsidized housing in NYC. Preventive Medicine, 185, 108023. https://doi.org/10.1016/j.ypmed.2024.108023](https://doi.org/10.1016/j.ypmed.2024.108023) | 0 | 0 | N |
| [Vesper, S., Robins, T., Lewis, T., Dombkowski, K., Wymer, L., Villegas, R., & Batterman, S. (2017). Use of Medicaid and housing data may help target areas of high asthma prevalence. Journal of Asthma, 54(3), 230–238. https://doi.org/10.1080/02770903.2016.1212370](https://doi.org/10.1080/02770903.2016.1212370) | 0 | 0 | N |
| [Werthmann, D. W., Rabito, F. A., Adamkiewicz, G., Reponen, T., Calafat, A. M., Ospina, M., & Chew, G. L. (2024). Pesticide exposure and asthma morbidity in children residing in urban, multi-family housing. Journal of Exposure Science & Environmental Epidemiology, 34(2), 241–250. https://doi.org/10.1038/s41370-023-00524-2](https://doi.org/10.1038/s41370-023-00524-2) | 0 | 0 | N |
| [Wi, C.-I., Gauger, J., Bachman, M., Rand-Weaver, J., Krusemark, E., Ryu, E., King, K. S., Katusic, S. K., & Juhn, Y. J. (2016). Role of individual-housing–based socioeconomic status measure in relation to smoking status among late adolescents with asthma. Annals of Epidemiology, 26(7), 455–460. https://doi.org/10.1016/j.annepidem.2016.05.001](https://doi.org/10.1016/j.annepidem.2016.05.001) | 0 | 0 | N |
